# STARTING A GSA STUDENT CHAPTER WITH POSTGRADUATE STUDENTS IN THE UK: OPPORTUNITIES AND CHALLENGES

**DOI:** 10.1093/geroni/igae098.1324

**Published:** 2024-12-31

**Authors:** Dialechti Tsimpida

**Affiliations:** University of Southampton, Southampton, England, United Kingdom

## Abstract

As the global aging population continues to expand, there is a growing demand for professionals skilled in gerontology. To meet this need, the creation of Gerontological Society of America (GSA) Student Chapters within Higher Education Institutions (HEIs) has emerged as a pioneering initiative to support and cultivate emerging gerontology professionals. The University of Southampton, home to the UK’s sole Department of Gerontology, was invited to establish a GSA Student Chapter in the 2023-24 academic year. While enthusiastic about the opportunity to deepen student engagement with the GSA, initiating a GSA Student Chapter within a postgraduate-focused program posed challenges. This endeavor involved strategic engagement efforts to recruit postgraduate students specializing in aging studies, and to organize activities to establish and formally affiliate the new Student Chapter with the University of Southampton. The initiative aimed to foster professional development in education, research, advocacy, policy, and sciences related to aging. Affiliation with GSA undoubtedly enriches the careers of postgraduate students by providing access to a global network of experts, mentorship opportunities, international connections, and a comprehensive toolkit for aging studies. In this session, Dr Dalia Tsimpida, Faculty Advisor of the newly established GSA Student Chapter at the University of Southampton, will discuss the rewarding experiences and challenges encountered in launching and establishing the Student Chapter. Attendees will be prompted to consider why and how a GSA Student Chapter can effectively engage postgraduate students specializing in aging studies, and how such chapters can be integrated into their respective Institutions.

